# The Quality of Fundamental Care as Perceived by Nurses and Patients in a Hospital Setting: A Descriptive Comparative Study

**DOI:** 10.1002/nop2.70234

**Published:** 2025-04-22

**Authors:** Hanna‐Sisko Kaukkila, Laura‐Maria Peltonen, Anna‐Sofia Korteniemi, Miko Pasanen, Sanna Salanterä, Pirjo Sibakov, Asta Heikkilä

**Affiliations:** ^1^ Turku University Hospital Turku Finland; ^2^ University of Turku Department of Nursing Science Turku Finland; ^3^ The Wellbeing Services County of Southwest Finland Research Services Turku Finland

**Keywords:** fundamental care, nurses, nursing, patient, quality

## Abstract

**Aim:**

To describe and compare nurses' perceptions of the quality of fundamental care and related factors before and after a clinical development project, and to describe patients' perceptions of the quality of fundamental care.

**Design:**

A descriptive, comparative study design.

**Methods:**

Data were collected online from nurses in 2015 and 2021 with a structured questionnaire exploring fundamental care through 12 areas at the beginning and the end of a clinical developmental project. Patient experiences were collected with a paper‐based questionnaire in 2021. A STROBE checklist was used as the reporting guideline.

**Results:**

The nurses assessed the overall quality of fundamental care as high at both data collection timepoints. However, the quality deteriorated in general and in specific areas, including skin condition and cleansing, elimination, nutrition and blood circulation, during the follow‐up period according to the nurses' experiences. A correlation was found between the areas of the quality of fundamental care. The registered nurses and the nurse managers expressed that not all areas of fundamental care were part of their work. The patients' experiences were mostly good. A small number of patients expressed a need to deal with issues related to their wellness of mind during their care.

**Conclusions:**

The quality of fundamental care was perceived as high by nurses and patients, but the quality deteriorated during the follow‐up period. It may be that the Covid‐19 pandemic and a general shortage of nurses in the hospital contributed to nurses having to decide how to prioritise their tasks between fundamental and specialised care. It is therefore recommended to further develop fundamental care in the hospital.

**Relevance to Clinical Practice:**

Nursing roles and practices need to be reviewed and developed further to better support nurses in their work to provide high‐quality fundamental care. A systematic evaluation of the quality of fundamental care is recommended.

**Patient or Public Contribution:**

Patients responded to the research questionnaire.

## 1 Introduction

Fundamental care is a central part of nurses' work in all healthcare settings (Kitson et al. 2010; Englebright et al. 2014; Feo et al. 2018) typically involving both patients and their family members (Kitson et al. 2010; Englebright et al. 2014). Nurses deliver fundamental care independently, without the need for medical orders from physicians (Englebright et al. 2014). However, the provision of high‐quality fundamental care requires collaboration among various healthcare professionals, ensuring the effective use of their expertise in the patient's overall care (Kitson and Muntlin Athlin 2013).

Quality of care refers to the extent to which the health services provided to patients increase the likelihood of the desired health outcomes (World Health Organization 2024). Fundamental care is a crucial part of health care, and it can significantly impact the survival, quality of life, well‐being and health outcomes of patients (Conroy et al. 2023; Ottonello et al. 2023) including patient recovery and length of stay (de Foubert et al. 2021). It also impacts safety, efficiency of health services and healthcare costs (Pentecost et al. 2020; de Foubert et al. 2021). This study focuses on the quality of fundamental care by examining the perceptions of nurses and patients.

## 2 Background

The concept and components of fundamental care have been defined in different ways in the literature (Kitson et al. 2010; Englebright et al. 2014; Feo et al. 2018; Pentecost et al. 2020; Ottonello et al. 2023). Ottonello et al. (2023) state that the term *fundamental care* has evolved over time and builds on an initial definition by Kitson et al. (2010). Feo et al. (2018) define fundamental care as nursing actions that respect and focus on a person's essential needs such as safety, respiration, eating and drinking, toileting, personal cleaning and dressing, mobility, rest and sleep, comfort (including pain management), communication, being involved and informed, dignity, respect and privacy to ensure a person's physical and psychosocial well‐being. There seems to be a broad consensus on the essential needs of the individual (Kitson et al. 2010; Feo et al. 2018). Fundamental care requires a patient‐centred approach, a trusting patient–nurse relationship, and continuous assessment of both the person's needs and the nursing activities (Conroy et al. 2023).

In their literature review, Ottonello et al. (2023) emphasise the importance of communication, the contextual environment, leadership influence and nurse–patient relationship in providing effective and high‐quality fundamental care. The elements of fundamental care are essential in providing not only quality care but also safe care to patients (Kitson et al. 2010). However, these elements may be underestimated by nursing professionals if they are merely seen as simple tasks (Feo et al. 2019). In addition, increasingly sicker patients and shorter treatment times, as well as an increasing emphasis on specialised care, can easily leave fundamental care less acknowledged and valued (Ausserhofer et al. 2014). The reason for failing to provide fundamental care is not necessarily due to devaluation of these tasks or a lack of attention related to the delivery of care, but rather a lack of support. Failure to deliver fundamental care can happen when nurses are overloaded with work (Ausserhofer et al. 2014), when the work environment is inconducive to effective fundamental care, when there is a shortage in staff or time (Ball et al. 2016), or as a result of a health crisis (Nagel et al. 2022; Sihvola et al. 2023). Nurses may also miss performing certain tasks of fundamental care when they use their work time to carry out non‐nursing tasks (Ausserhofer et al. 2014). Nursing leaders have the responsibility of ensuring that the essential needs of nurses are met and that patient outcomes are achieved through a coordinated and collaborative process within the nursing team (Mudd et al. 2023; Ottonello et al. 2023). Nurse leaders have the potential to enhance nursing staff satisfaction and positively influence the quality of care and patient outcomes through a transformational leadership approach (Robbins and Davidhizar 2020; Gebreheat et al. 2023). However, to fully realise this potential, nursing leaders require the support of healthcare organisations to manage the demanding leadership responsibilities (Cox 2019).

Nurses may prioritise and deliver care differently depending on their tasks and working environment (Kitson et al. 2013). In Finland, fundamental care is the area of expertise of practical nurses (PNs) and an important part of registered nurses' (RNs) work as well. One big difference between a PN and a RN is their level of education and training. PN education includes three years of vocational education (180 competence points). PNs can work within healthcare or social care with a protected occupational title (The Finnish Union of Practical Nurses 2023). The length of the bachelors'‐level nursing education is 3.5 years (210 credits). This degree enables qualification as a registered nurse. A bachelor's degree in nursing is granted by universities of applied sciences, and the education has a competency‐based approach as defined by the European Union directives. After receiving a bachelor's degree, RNs can continue to master's studies to specialise in, for example, nursing management and leadership (Salminen et al. 2019.) Nurse managers (NMs) management and leadership activities focus on recruitment, organising, work well‐being, work atmosphere, communication, clinical nursing, development of the unit, personnel development, development of nursing, financial management, planning and evaluation of activities and development with collaborating partners (Nurmeksela 2021; Nurmeksela et al. 2021). The emphasis on tasks of NMs varies in practice. According to the study by Nurmeksela et al. (2021), organising and developing the unit were the most frequently performed activities, while clinical work was the least common.

Previous studies on fundamental care have explored its conceptual underpinnings (Ottonello et al. 2023), its components (Jangland et al. 2018), factors influencing its delivery (Conroy 2018), the supportive role of nurse managers in its delivery (Mudd et al. 2023), the experiences of patients, residents and nurses (Pentecost et al. 2020) and nursing interventions related to basic human needs (Richards et al. 2018). Moreover, van Belle et al. (2020) explored how nurses in hospitals implement person‐centred fundamental care. However, fundamental care practices are not always based on research evidence (Richards et al. 2018) and prior studies have found a lack in its implementation, for example, related to hygiene (e.g., Ausserhofer et al. 2014) and mobility (e.g., Grealish et al. 2023). In a review by Pentecost et al. (2020), most of the included 47 studies considered hygiene and mobility, and only a few of the studies examined elimination and nutrition. Research also shows that nurses (van Belle et al. 2020) and nursing students (Jangland et al. 2018) mainly identify and focus on the physical care needs of patients and concentrate less on the psychosocial and interpersonal needs. NMs, on the other hand, are perceived to insufficiently support staff in providing fundamental care (Mudd et al. 2023).

Most studies related to fundamental care have focused on observational or physiological measures, with very few collecting patient‐reported outcomes, such as quality of life, experience or self‐reported symptoms (Richards et al. 2018). Although studies have been published about the components of fundamental care, some of these have been found to be of poor quality (Richards et al. 2018; Pentecost et al. 2020). More research is therefore needed in different types of care settings, both in acute and long‐term care facilities, and among patients of different ages (Savoie et al. 2023). There is also a need for more coordinated collaboration and research in fundamental care between all members of the healthcare team (Kitson et al. 2019). Gaps in research related to the quality of fundamental care make it difficult to develop evidence‐based practices and assess the quality of fundamental care (Richards et al. 2018; Pentecost et al. 2020).

In an attempt to improve the quality of fundamental care, a Finnish university hospital implemented a clinical development project called ‘Health‐producing fundamental care using smart technologies’ (hereafter STEPPI) between 2016 and 2020. The goal of this project was to develop and implement evidence‐based and high‐quality fundamental care by guiding the development of nursing activities in a practical, step‐by‐step manner. Fundamental care is a key area in the assessment of effectiveness and quality of care (de Foubert et al. 2021). In addition to collecting data on nurses' assessment of the care they provide to patients, patient feedback has become a valuable information source, because it reflects the successes and failures of care (Richards et al. 2023). Developing the quality of fundamental care is an ongoing process. Hence, it is important to collect information on the achieved results to continuously improve care.

## 3 Methods

### 3.1 Aim

The aim of this study was twofold. First, it aimed to describe and compare nurses' perceptions of the quality of fundamental care and related factors before and after a clinical development project (STEPPI). Second, it aimed to describe patients' perceptions of the quality of fundamental care. In this study, the term *nurse* includes PNs, RNs and NMs.

### 3.2 Design and Setting

This study used a descriptive, comparative study design with two data collection time points. Data were collected via questionnaires at one public university hospital in southern Finland in the autumn of 2015 and in the spring of 2021. In the autumn of 2015, the survey evaluating the quality of fundamental care was distributed to nursing staff. After this, the STEPPI project was initiated. In this project, a set of twelve minimum criteria were defined based on the research literature on fundamental care. These criteria were implemented into each hospital unit with the help of *project agents*, whose role was to promote the development of fundamental care together with the work community, based on the unit's objectives and needs. The minimum criteria were research‐based, general target levels of quality of fundamental care, which units' could adapt as needed. The aim of the minimum criteria was to help nurses better understand what fundamental care entails and how it can be recognised and systematically implemented into practice. The project agents familiarised themselves with fundamental care and its components and briefed other staff in the units by sharing information and organising workshops. Areas for improvement were identified by comparing current practices with the minimum criteria. The project agents documented the development objectives and methods in workbooks.

In addition, the STEPPI project has played a central role in the development of patient documentation and in strengthening the competency of nurses. Thus, in addition to unit level activities, project facilitators organised hospital‐level joint training, workshops and online courses on topics related to various medical specialties, such as hand hygiene and aseptic processes, the assessment of the vascular cannula injection site to prevent infections, pain management, malnutrition prevention, the prevention of falls, the prevention of pressure ulcers and the management of evidence‐based nursing. The project was designed to run for five years, as it commonly takes much time and resources to bring about change in a large organisation. STEPPI emphasised a bottom‐up approach, with nurses themselves identifying the areas in need of improvement. The Covid‐19 pandemic, which spread to the region in 2020, did not impede the project as fundamental care was recognised as increasingly important and timely in the care of critically ill patients. The project progressed according to the capacity of the units and was supported by online meetings, among other things. In the spring of 2021, a follow‐up survey was carried out with the nursing staff. It was also considered important to study patients' experiences of fundamental care as the hospital was aiming to strategically further develop patient satisfaction and its evaluation. Patient feedback is a valuable source of information in evaluating the quality of care (Richards et al. 2023). The reporting of this study followed the Strengthening the Reporting of Observational Studies in Epidemiology (STROBE) guidelines.

The study was carried out in a Finnish university hospital, which produces extensive specialised healthcare services. More than 250,000 people use the hospital's services annually, and this number has been increasing during the last years. The average length of stay in the somatic inpatient wards is 3.1 days (Turku University Hospital, Tyks 2023). Overall, patients have reported being satisfied with the care provided at the hospital. In 2022, 95.2% of patients assessed the care they received as good (Tyks 2023). Around 400 PNs, 3000 RNs and 240 NMs (including assistant NMs) are employed at the hospital, and the numbers have remained approximately the same during the last years. However, the turnover of permanent staff has been increasing (Tyks 2023).

### 3.3 Data Collection

The data collection was targeted at nurses working in various medical specialties during the initial phase of the project (in October–November 2015, *N* = approximately 2500) and after the implementation of the project (in May 2021, *N* = 2416). The inclusion criteria for nurses were as follows: (1) a job title of PN, RN or NM, (2) working in a medical specialty that provides direct patient care, and (3) working in an inpatient unit, an outpatient unit, an operating department, a critical care unit or an emergency department. Nurses working in radiology and radiological imaging or in a laboratory were excluded. The data were collected via an electronic questionnaire (Webropol software). The NMs sent out an invitation to nursing staff in all units to participate in the survey as well as a link to the questionnaire. Nurses completed the questionnaire at a time of their choosing. Responding took approximately 10–20 min.

Patient experiences regarding the received fundamental care were collected from adult patients in May 2021 in seven medical specialities using a paper‐based questionnaire. The target group (*N* = 384) consisted of all patients in the inpatient wards (*n* = 29) who were able to give informed consent for participation in the study and complete the questionnaire. Each ward was able to choose the most appropriate time for data collection during a specified week in May. Patients completed the questionnaire anonymously and returned it by placing it into a secured box at the nursing office in their unit. The questionnaire was completed anonymously and took approximately 5–10 min to complete. Contact persons from each medical speciality coordinated and guided the practical arrangements for the data collection.

### 3.4 Questionnaires

Data from the nursing staff were collected through the Quality of Fundamental Care questionnaire to address the self‐reported quality of fundamental care and the documentation of care. This questionnaire was developed based on the components of fundamental care (Kitson et al. 2010, 2013), guidance and classification on the nursing documentation in Finland (Liljamo et al. 2012), and discussion with the hospital's nursing development team, consisting of nurse researchers, nursing directors and clinical nurse specialists, in 2015. The questionnaire included 42 items designed to address the assessment, meeting (i.e., supporting, enabling), documentation and compliance of the 12 areas of fundamental care: skin condition and cleansing (3 items), personal hygiene and professionals' aseptic behaviour (2 items), elimination (3 items), oral hygiene (3 items), nutrition (5 items), nausea (3 items), mobility and postural care (5 items), blood circulation (3 items), respiration (3 items), rest and sleep (5 items), pain (4 items) and wellness of mind (3 items). In 2021, the questionnaire items were linguistically revised (e.g., word order) to clarify readability. Moreover, a few items were split into two, as they contained two issues (e.g., ‘I assess the patient's need for mobility and assistive devices and guide the patient according to their condition’). After these revisions, the total number of items was 50. The changes were made so that the areas of fundamental care remained comparable in the data sets of both data collection years.

The hospital's nursing development team, consisting of clinical nurse specialists, NMs, nurse directors and university researchers (*n* = 22), evaluated the content validity of the questionnaire by assessing the appropriateness of the items and the language used. Based on these assessments, the linguistic expression of a few items was clarified. The Cronbach's alpha coefficient was used to test the consistency of the questionnaire. The internal consistency of the areas of the instrument ranged between 0.58 and 0.92 in 2015 and between 0.62 and 0.90 in 2021. Nurses were asked to rate the quality of the fundamental care they provided in their daily work on a scale of 4–10, which is widely used in the Finnish educational system (4 = weak, 10 = excellent). The respondent could select the option 0 if the care activity item was not part of their work. In addition, the questionnaire included three items on characteristics of the respondent, including job title, type of unit and medical speciality in which the respondent worked.

Patient data were collected using the Patient Care Performance Measure questionnaire, which was derived from the Quality of Fundamental Care questionnaire described above. This questionnaire captured patients' experiences of the quality of fundamental care through one item for each of the 12 areas of fundamental care (i.e., ‘I got enough food and drink considering possible special dietary needs’). Respondents answered the items (*n* = 12) with ‘yes’, ‘no’ or ‘not applicable to me’. The questionnaire was deliberately kept short to promote completion. The intent was to capture the patients' general views on the implementation of each area of fundamental care rather than the specific issues within each area. In addition, patients were asked to respond to two open‐ended items: ‘Insights on the previous questions’ and ‘How do you think the patient care should be improved?’ Characteristics collected from patient respondents included medical speciality and inpatient ward. The hospital's nursing development team and a few people close to the team members reviewed the content of the questionnaire, which led to clarifications in the language used in some of the items.

### 3.5 Ethical Considerations

Ethical principles were followed throughout the study. The ethics committee of the University of Turku approved the study plan (reg. no. 36/2020). In addition, research permission was obtained from the university hospital. All participants gave written informed consent. To protect the anonymity of the participants, they were not asked for personal information. Data were managed confidentially. The results were made available to the nursing directors and NMs only if there were at least 10 responses in the unit. The results were discussed with the staff in a spirit of encouragement and continuous improvement.

### 3.6 Data Analysis

The data collected from the nurses were statistically analysed using the R software version 4.0.2. Sums were formed for the whole scale and its subscales excluding the response ‘not part of my work’. Participants with ≥ 30% missing values were excluded from the analysis to control for bias due to respondents who answered ‘not part of my work’ for most items. Descriptive statistics were calculated for the respondent characteristics and sums. The reliability of the scale and its subscales were assessed by Cronbach's alpha coefficient for both data sets. The correlations of the subscales were assessed using the Spearman's correlation coefficient. Differences in sums between the years were tested using the Mann–Whitney *U* test. Associations between the characteristics and sum variables were tested using the Kruskal–Wallis test. The Dunn's test with Bonferroni correction was used for post hoc testing. For questions, where at least 25% of the respondents had answered ‘not part of my work’, the Chi‐square test was used to determine whether there was a relationship between the background variables and the responses. A statistical test was considered significant if the *p* value was ≤ 0.05.

The quantitative data collected from the patients were analysed with SPSS 27.0 by calculating frequencies and percentages. Responses to the open‐ended questions were analysed using content analysis (Graneheim and Lundman 2004). Two researchers carried out the analysis. The open‐ended questions were written in a word file and read several times to gain a clear overall picture. The responses were grouped into categories, which were formed inductively based on similarities, and frequencies were calculated.

## 4 Results

### 4.1 Characteristics of Nurses and Patients

A total of 546 nurses responded to the survey in 2015 (response rate 21.8%) and 468 nurses in 2021 (response rate 19.4%). In 2015, the respondents included 107 PNs, 397 RNs, 38 NMs and four who reported their role as ‘other’. In 2015, 336 of the respondents worked on inpatient wards while 209 were affiliated with other units (i.e., outpatient units, operating departments, critical care units and emergency department) and one respondent failed to report a unit. In 2021, the respondents included 80 PNs, 361 RNs, 25 NMs and two who did not reported their role. In 2021, 276 respondents reported working in inpatient wards, 190 reported working in other units and two did not to report a unit. A total of 274 patients (response rate 71.4%) from seven medical specialities and 29 inpatient wards responded to the questionnaire.

### 4.2 Quality of Fundamental Care and Associations Between Areas as Reported by Nurses in 2015 and 2021

Overall, the reported mean for the quality of fundamental care in the hospital was 8.94 (SD 0.79) in 2015 and 8.78 (SD 0.78) in 2021. There was a slight decrease in the reported quality of fundamental care (difference −0.19, *p* = 0.001) between the years 2015 and 2021. The highest area of fundamental care rated by nurses in both 2015 and 2021 was the area related to elimination (mean 9.47 vs. 9.23, SD 0.71 vs. 1.06, difference −0.24, *p* = 0.001) and the lowest area in both years was oral hygiene (mean 8.08 vs. 8.13, SD 1.35 vs. 1.32, difference 0.05, *p* = 0.572) (Table 1).

**TABLE 1:** Quality of fundamental care reported by nurses in 2015 and 2021.

Areas of fundamental care	2015			2021			Difference	*p* *
	*n*	Mean	SD	*n*	Mean	SD		
1. Skin condition and cleansing	471	9.18	0.87	370	8.7	1.13	−0.48	< 0.001
2. Personal hygiene and professionals' aseptic behaviour	530	9.22	0.84	445	9.08	0.97	−0.14	0.097
3. Elimination	501	9.47	0.71	404	9.23	1.06	−0.24	0.001
4. Oral hygiene	428	8.08	1.35	371	8.13	1.32	0.05	0.572
5. Nutrition	484	8.75	0.92	414	8.52	1.06	−0.23	0,004
6. Nausea	505	9.15	0.89	417	8.89	1.01	−0.26	< 0.001
7. Mobility and postural care	464	8.36	1.16	406	8.64	1.04	0.28	< 0.001
8. Blood circulation	511	9.37	0.8	421	8.92	1.09	−0.45	< 0.001
9. Respiration	493	9.32	0.89	408	9.11	1.07	−0.21	0.007
10. Rest and sleep	480	8.94	0.98	420	8.8	1.09	−0.14	0.113
11. Pain	495	8.6	1.15	419	8.81	1.04	0.21	0.006
12. Wellness of mind	527	9.03	1.04	444	9	0.97	−0.03	0.343
Overall fundamental care	470	8.94	0.79	389	8.78	0.78	−0.19	0.001

Abbreviation: SD, Standard deviation.

* Mann–Whitney *U* test.

**FIGURE 1:**
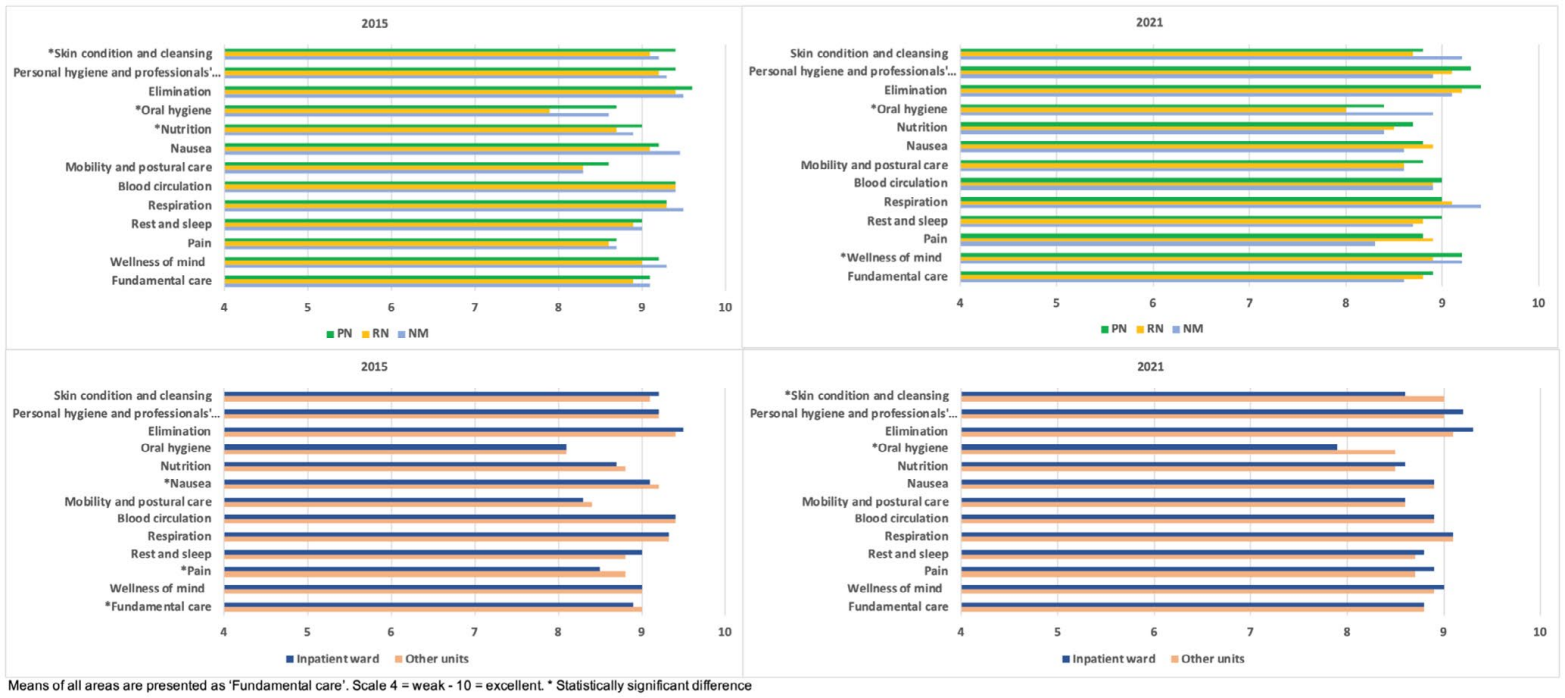
Reported quality of fundamental care by role and unit presented for 2015 and 2021.

Differences in the reported quality of fundamental care based on the nurses' role and unit somewhat differed by area and year (Figure 1). A difference by role was seen in 2015 in the sum variable of fundamental care (*p* < 0.05) and in the areas of skin condition and cleansing (*p* < 0.05), oral hygiene (*p* < 0.001) and nutrition (*p* < 0.05). A more detailed analysis showed that PNs reported higher values than RNs in the areas of skin condition and cleansing (mean 9.4 vs. 9.1, SD 0.66 vs. 0.92, *p* < 0.05), oral hygiene (mean 8.7 vs. 7.9, SD 1.12 vs. 1.37, *p* < 0.001) and nutrition (mean 9.0 vs. 8.7, SD 0.81 vs. 0.94, *p* < 0.05). In addition, NMs reported higher values than RNs regarding the area of oral hygiene (mean 7.9 vs. 8.6, SD 1.37 vs. 1.15, *p* < 0.05). However, no difference by role was found in the sum variable of fundamental care. In 2021, a difference by role was seen in the areas of oral hygiene (*p* < 0.05) and wellness of mind (*p* < 0.05). A more detailed analysis showed that PNs reported higher values than RNs in the areas of oral hygiene (mean 8.4 vs. 8.0, SD 1.23 vs. 1.34, *p* < 0.05) and wellness of mind (mean 9.2 vs. 8.9, SD 0.9 vs. 1.0, *p* < 0.05). In 2015, compared to those working in inpatient wards, respondents working in other units reported higher values for the sum variable of fundamental care (mean 8.9 vs. 9.0, SD 0.73 vs. 0.88, *p* < 0.05) as well as in the areas of nausea (mean 9.1 vs. 9.2, SD 0.82 vs. 1.0, *p* < 0.05) and pain (mean 8.5 vs. 8.8, SD 1.14 vs. 1.16, *p* = 0.001). In 2021, compared to those working in inpatient wards, nurses working in other units reported higher values in the areas of skin condition and cleansing (mean 8.6 vs. 9.0, SD 1.12 vs. 1.12, *p* < 0.001) and oral hygiene (mean 7.9 vs. 8.5, SD 1.27 vs. 1.33, *p* < 0.001).

The various areas of fundamental care are interconnected (*r* ≥ 0.3, *p* < 0.001) as shown in Table 2. However, the correlation scores generally decreased per area between the years 2015 and 2021. The lowest correlations (< 0.4) were observed in 2021. The highest correlation showed a moderate positive relationship between the areas of respiration and blood circulation in both 2015 (*r* = 0.77) and 2021 (*r* = 0.71), and mobility and postural care and blood circulation in 2021 (*r* = 0.71).

**TABLE 2:** Correlations between areas of reported quality of fundamental care.

Areas of fundamental care	2015 vs. 2021	2015 vs. 2021	2015 vs. 2021	2015 vs. 2021	2015 vs. 2021	2015 vs. 2021	2015 vs. 2021	2015 vs. 2021	2015 vs. 2021	2015 vs. 2021	2015 vs. 2021	2015 vs. 2021
	Skin condition and cleansing	Personal hygiene and professionals’ aseptic behaviour	Elimination	Oral hygiene	Nutrition	Nausea	Mobility and postural care	Circulation	Respiration	Rest and sleep	Pain	Wellness of mind
Skin condition and cleansing	1											
Personal hygiene and professionals’ aseptic behaviour	0.60 vs. 0.48	1										
Elimination	0.64 vs. 0.48	0.58 vs. 0.48	1									
Oral hygiene	0.53 vs. 0.62	0.51 vs. 0.44	0.45 vs. 0.40	1								
Nutrition	0.54 vs. 0.45	0.51 vs. 0.55	0.54 vs. 0.44	0.59 vs. 0.46	1							
Nausea	0.57 vs. 0.39	0.53 vs. 0.37	0.63 vs. 0.46	0.52 vs. 0.40	0.61 vs. 0.53	1						
Mobility and postural care	0.49 vs. 0.50	0.45 vs. 0.51	0.46 vs. 0.51	0.50 vs. 0.51	0.66 vs. 0.57	0.53 vs. 0.55	1					
Blood circulation	0.59 vs. 0.50	0.53 vs. 0.50	0.64 vs. 0.55	0.46 vs. 0.48	0.60 vs. 0.57	0.65 vs. 0.55	0.53 vs. 0.71	1				
Respiration	0.57 vs. 0.52	0.44 vs. 0.44	0.62 vs. 0.55	0.44 vs. 0.40	0.58 vs. 0.44	0.59 vs. 0.49	0.50 vs. 0.59	0.77 vs. 0.71	1			
Rest and sleep	0.49 vs. 0.34	0.45 vs. 0.36	0.54 vs. 0.43	0.50 vs. 0.32	0.55 vs. 0.39	0.60 vs. 0.44	0.59 vs. 0.49	0.60 vs. 0.50	0.62 vs. 0.50	1		
Pain	0.40 vs. 0.36	0.44 vs. 0.48	0.43 vs. 0.44	0.41 vs. 0.35	0.52 vs. 0.50	0.54 vs. 0.58	0.55 vs. 0.56	0.53 vs. 0.58	0.52 vs. 0.49	0.53 vs. 0.44	1	
Wellness of mind	0.51 vs. 0.34	0.42 vs. 0.31	0.52 vs. 0.37	0.44 vs. 0.30	0.52 vs. 0.36	0.53 vs. 0.36	0.48 vs. 0.43	0.56 vs. 0.40	0.52 vs. 0.39	0.66 vs. 0,61	0.49 vs. 0.40	1
Overall fundamental care	0.69 vs. 0.69	0.66 vs. 0.68	0.71 vs. 0.69	0.70 vs. 0.67	0.80 vs. 0.71	0.78 vs. 0.71	0.81 vs. 0.81	0.77 vs. 0.83	0.75 vs. 0.77	0.79 vs. 0.63	0.75 vs. 0.79	0.71 vs. 0.56

*Note:* All correlations were statistically significant (*p* ≤ 0.05).

### 4.3 Fundamental Care Reported by Nurses as ‘Not Part of My Work’ by Role and Unit

There were several items that were reported as ‘not part of my work’ by the respondents. The 10 most reported items were reported by 22.2%–37.8% of the respondents in 2015 and 25.0%–41.7% of the respondents in 2021. In both years, the three most reported items were related to the same areas of fundamental care: mobility and postural care, oral hygiene and nutrition. However, the order of these items varied. In both years, two of these items concerned the use of risk assessment tools. The top 10 least reported items were identified by 4.2%–9.4% of the respondents in 2015 and 5.2%–12.4% in 2021. The three least reported items were related to the area of wellness of mind and the order was the same in both years (Table 3).

**TABLE 3:** Top 10 most and least reported items ‘not part of my work’ to nurses in 2015 and in 2021.

Top 10 most reported items ‘not part of my work’ to nurses			
2015	%	2021	%
Using of the pressure injures risk assessment instrument (Area 7)	37.80	Using of a nutrition risk assessment instrument (Area 5)	41.72
Enabling the patient to wear dentures (Area 4)	29.87	Using of the pressure injures risk assessment instrument (Area 7)	38.58
Using of a nutrition risk assessment instrument (Area 5)	29.65	Caring for the patient's dentures (Area 4)	30.89
Providing and implementing postural care for a patient every 2 h (Area 7)	28.70	Caring and guidance of mouth and mucous membranes (Area 4)	29.18
Caring for the patient's oral health (Area 4)	28.60	Providing and implementing postural care for a patient every 4 h (Area 7)	28.26
Providing and implementing postural care for a patient every 4 h (Area 7)	28.10	Enabling patients to wear dentures (Area 4)	28.14
Assessment of patient nutritional supplement need and giving them (Area 5)	26.25	Providing and implementing postural care for a patient every 2 h (Area 7)	26.94
Ensuring good cleanliness for the patient during care (Area 1)	25.18	Guiding the patient to get moving as early as possible (Area 8)	26.84
Documenting of patient's oral and mucosal care (Area 4)	24.26	Documenting of patient oral and mucosal condition and treatment (Area 4)	25.75
Providing as peaceful sleeping environment as possible for the patient (Area 10)	22.22	Assessment of patient nutritional supplement need and giving them (Area 5)	25.05

Top 10 least reported items ‘not part of my work’ to nurses			
2015	%	2021	%
Assessment of patient's wellness of mind (Area 12)	4.22	Assessment of patient's wellness of mind (Area 12)	5.15
Supporting patient's wellness of mind (Area 12)	4.41	Supporting patient's wellness of mind (Area 12)	5.36
Documenting assessment of patient wellness of mind (Area 12)	5.33	Documenting assessment of patient wellness of mind (Area 12)	6.21
Assessment of patient blood circulation (Area 8)	5.92	Compliance with aseptic instructions (Area 2)	7.08
Documenting the assessment of patient blood circulation (Area 8)	6.09	Assessment of patient blood circulation (Area 8)	8.79
Documenting the assessment of patient nausea (Area 6)	7.21	Documenting the assessment of patient blood circulation (Area 8)	10.58
Assessment of patient nausea (Area 6)	7.41	Documenting patient drug treatment (Area 11)	11.15
Assessment of patient respiration (Area 9)	8.49	Documenting patient non‐drug treatment (Area 11)	11.30
Documenting patient respiratory monitoring(Area 9)	8.53	Documenting the assessment of patient nausea (Area 6)	12.20
Documenting patient pain treatment methods(Area 11)	9.44	Assessment of the patient's respiration (Area 9)	12.39

There was a statistically significant difference in responses given by nurses in different roles and units regarding the items of fundamental care perceived as ‘not part of my work’. In general, NMs reported more items as ‘not part of my job’ than PNs. There was a 28% increase in items reported as ‘not part of my work’ from 2015 to 2021. Two items stood out in the responses with higher frequencies: the need to use risk assessment instruments for pressure injuries in 2015 (Area 7) and malnutrition in 2021 (Area 5) (Table 4).

**TABLE 4:** Items of fundamental care reported as ‘not part of my work’ that changed statistically significantly between the years 2015 and 2021.

2015	Items										
	Ensuring good cleanliness for the patient during care (Area 1)	Caring and guidance of mouth and mucous membranes (Area 4)	Enabling patients to wear dentures (Area 4)		Using of a nutrition risk assessment instrument (Area 5)	Assessment of nutritional supplement need and giving them (Area 5)		Providing and implementing postural care for a patient every 4 h (Area 7)	Using of the pressure injures risk assessment instrument (Area 7)		
Role											
*p* *	*p* < 0.001	*p* < 0.001	*p* < 0.001		*p* < 0.001	*p* < 0.001		*p* < 0.001	*p* = 0.001		
PN	4.67% (*n* = 5)	4.71% (*n* = 5)	13.46% (*n* = 14)		17.92% (*n* = 19)	8.57% (*n* = 9)		8.49% (*n* = 9)	24.04% (*n* = 25)		
RN	27.34% (*n* = 108)	32.23% (*n* = 127)	32.31% (*n* = 127)		30.37% (*n* = 120)	28.17% (*n* = 111)		30.27% (*n* = 119)	39.39% (*n* = 154)		
NM	55.26% (*n* = 21)	52.63% (*n* = 20)	44.73% (*n* = 17)		50% (*n* = 19)	52.63% (*n* = 20)		55.26% (*n* = 21)	55.26% (*n* = 21)		
Unit											
*p* **	*p* < 0.001	*p* < 0.001	*p* < 0.001		*p* < 0.001	*p* < 0.001		*p* < 0.001	*p* < 0.001		
Inpatient ward	2.68% (*n* = 9)	4.50% (*n* = 15)	12.39% (*n* = 41)		10.18% (*n* = 34)	4.78% (*n* = 16)		7.51% (*n* = 25)	18.60% (*n* = 61)		
Other	61.35% (*n* = 127)	66.82% (*n* = 139)	57.49% (*n* = 119)		61.06% (*n* = 127)	61.46% (*n* = 126)		60.87% (*n* = 126)	67.79% (*n* = 141)		

2021	Items										
	Ensuring good cleanliness for the patient during care (Area 1)	Care and guidance of mouth and mucous membranes (Area 4)	Enabling patients to wear dentures (Area 4)	Caring for the patient's dentures (Area 4)	Using of a nutrition risk assessment instrument (Area 5)	Assessment of nutritional supplement need and giving them (Area 5)	Assessing the patient's mobility and the need for assistive devices (Area 7)	Providing and implementing postural care for a patient every 4 h (Area 7)	Using of the pressure injures risk assessment instrument (Area 7)	Providing and implementing postural care for a patient every 2 h (Area 7)	Guiding the patient to get moving as early as possible (Area 8)
Role											
*p* *	*p* < 0.001	*p* < 0.001	*p* = 0.002	*p* < 0.001	*p* = 0.016	*p* < 0.001	*p* < 0.001	*p* < 0.001	*p* = 0.006	*p* < 0.001	*p* < 0.001
PN	6.25% (*n* = 5)	10.12% (*n* = 8)	16.67% (*n* = 13)	16.67% (*n* = 13)	29.11% (*n* = 23)	10.12% (*n* = 8)	1.28% (*n* = 1)	16.25% (*n* = 13)	27.50% (*n* = 22)	16.67% (*n* = 13)	15.19% (*n* = 12)
RN	26.04% (*n* = 94)	31.02% (*n* = 112)	28.85% (*n* = 103)	31.84% (*n* = 114)	43.33% (*n* = 156)	26.94% (*n* = 97)	15.77% (*n* = 56)	28.08% (*n* = 104)	39.38% (*n* = 141)	26.94% (*n* = 97)	27.45% (*n* = 98)
NM	60% (*n* = 15)	62.50% (*n* = 15)	52% (*n* = 13)	60% (*n* = 15)	58.33% (*n* = 14)	45.45% (*n* = 10)	36.00% (*n* = 9)	58.33% (*n* = 14)	62.50% (*n* = 15)	58.33% (*n* = 14)	56% (*n* = 14)
Unit											
*p* **	*p* < 0.001	*p* < 0.001	*p* < 0.001	*p* < 0.001	*p* < 0.001	*p* < 0.001	*p* < 0.001	*p* < 0.001	*p* < 0.001	*p* < 0.001	*p* < 0.001
Inpatient ward	6.52% (*n* = 18)	9.82% (*n* = 27)	12.54% (*n* = 34)	14.02% (*n* = 38)	24.09% (*n* = 66)	6.18% (*n* = 17)	3.33% (*n* = 9)	11.60% (*n* = 32)	21.98% (*n* = 60)	12.45% (*n* = 34)	9.22% (*n* = 25)
Other	50.53% (*n* = 96)	57.14% (*n* = 108)	50.26% (*n* = 95)	54.74% (*n* = 104)	67.20% (*n* = 127)	52.69% (*n* = 98)	30.85% (*n* = 58)	52.38% (*n* = 99)	62.43% (*n* = 118)	47.62% (*n* = 90)	51.85% (*n* = 98)

Abbreviations: NM, nurse manager; PN, practical nurse; RN, registered nurse.

* Pearson's chi‐squared test.

** Fisher's exact test.

### 4.4 Patients' Experiences of the Quality of Fundamental Care

When looking at the patients' experiences of fundamental care per area, patients reported receiving the best guidance and assistance in relation to nutrition (92.5%), skin condition and cleansing (85.7%) and pain (85.4%). The patients felt they received the least guidance and assistance regarding their wellness of mind (11.2%), oral hygiene (8.3%) and personal hygiene and professionals' aseptic behaviour (6.7%) (Table 5).

**TABLE 5:** Patients' experiences of the quality of fundamental care.

Area of fundamental care	*n*	Yes % (*n*)	No % (*n*)	Not applicable to me % (*n*)
Nutrition (Area 5)	268	92.5 (248)	2.2 (6)	5.3 (14)
Skin condition and cleansing (Area 1)	266	85.7 (228)	3.4 (9)	10.9 (29)
Pain (Area 11)	267	85.4 (228)	4.1 (11)	10.5 (28)
Personal hygiene and professionals' aseptic behaviour(Area 2)	266	83.5 (222)	6.7 (18)	9.8 (26)
Rest and sleep (Area 10)	261	75.5 (197)	5 (13)	19.5 (51)
Elimination (Area 3)	267	74.9 (200)	5.2 (14)	19.9 (53)
Mobility and postural care (Area 7)	265	72.8 (193)	5.3 (14)	21.9 (58)
Oral hygiene (Area 4)	265	67.2 (178)	8.3 (22)	24.5 (65)
Blood circulation (Area 8)	265	64.9 (172)	6 (16)	29.1 (77)
Nausea (Area 6)	263	60.1 (158)	3.8 (10)	36.1 (95)
Respiration (Area 9)	264	57.6 (152)	6.4 (17)	36 (95)
Wellness of mind (Area 12)	259	47.5 (123)	11.2 (29)	41.3 (107)

Patients were asked to provide feedback on two open‐ended questions regarding the delivery of fundamental care, as well as suggestions for improving patient care. A total of 129 patients (response rate 47.1%) responded to these questions. The responses regarding the delivery of fundamental care were classified into four categories: (1) Providing professional and safe care, (2) Meeting physical and psychological needs, (3) Supporting social interaction and counselling, and (4) Enabling a health promoting care environment. Each of these categories included both positive and negative insights. Patients mostly reported good experiences within the areas of supporting social interaction and counselling (*n* = 65) and providing professional and safe care (*n* = 30). Twenty of the patients reported good experiences and 11 not so good experiences regarding how services met their physical and psychological needs (Table 6).

**TABLE 6:** Patients' (*n* = 129) insights of fundamental care and suggestions for improving it.

Questions	Categories	Number of insights
**Insights on fundamental care**	**Providing professional and safe care**	*n* = 30, of which positive insight = 30, negative insight = 0
	‘The ward has particularly good care—on a scale of 0–10 the ward gets 10+. Thanks to everyone for their care and professionalism’ (Lo84)	
	‘The work is done with heart and expertise. I felt safe. Thank you, you are top!’ (Lo92)	
	**Meeting the physical and psychological needs**	*n* = 31, of which positive insight = 20, negative insight = 11
	‘I have had enough food and drink and my diet has been taken into account perfectly’. (Lo180)	
	‘Several days of ineffective pain medication has been discussed on my behalf, but no changes are happening’. (Lo159)	
	**Supporting social interaction and counselling**	*n* = 71, of which positive insight = 65, negative insight = 6
	‘The staff have been friendly, helpful, understanding and have been able to listen to the patient in the neurology ward’. (Lo100)	
	‘Lab results and other test results are not always disclosed unless you ask. Similarly, the progress of treatment/investigation sometimes seems to depend on your asking’. (Lo159)	
	**Enabling a health promoting care environment**	*n* = 11, of which positive insight = 8, negative insight = 3
	‘The regular rhythm of the hospital provides reassurance and security for the patient during an unprecedented and worrying procedure’. (Lo113)	
	‘An alarm sounds at night at irregular times up to 16 times in a row’. (Lo75)	
**Suggestions on improving of fundamental care**	**Providing access to information**	*n* = 18
	‘I think it would be good if the nurse and the doctor explained a bit more about the treatment to the patient’. (Lo248)	
	**Providing patient‐centred care**	*n* = 17
	‘Listening to what the patient is feeling’. (Lo96)	
	**Ensuring privacy**	*n* = 3
	‘All personal matters were discussed loudly in front of the others’. (Lo25)	
	**Meeting the physical needs**	*n* = 10
	‘The patient needs to be listened to better about pain’. (Lo163)	
	**Ensuring conditions for high‐quality care**	*n* = 9
	‘All departments must have enough staff and their well‐being at work must be taken care of, as well as motivation and the maintenance of professional skills’. (Lo82)	

Responses on ways to improve patient care in the future were classified into five categories: (1) Providing access to information, (2) Providing patient‐centred care, (3) Ensuring privacy, (4) Meeting physical needs, and (5) Ensuring conditions for high‐quality care. Most suggestions for improvement were related to providing patients with access to information (*n* = 18) and providing patient‐centred care (*n* = 17) (Table 6). Of these 129 patients, 39 reported that there was nothing to improve, or that the care was already being developed in a positive direction. Moreover, seven patients had concerns about staff exhaustion.

## 5 Discussion

The aim of this study was twofold. First, it aimed to describe and compare nurses' perceptions of the quality of fundamental care and related factors before and after a clinical development project (STEPPI). Second, it aimed to describe patients' perceptions of the quality of fundamental care. The results indicate that the overall quality of fundamental care in the hospital was assessed as high by nurses in both 2015 and 2021. However, the reported quality deteriorated during the follow‐up period. The decrease in the quality of fundamental care overall and in specific areas, such as skin condition and cleansing, elimination, nutrition and blood circulation was statistically significant. This was not expected and is worrisome as the STEPPI project was planned to increase the quality of fundamental care. We can only speculate about the reasons behind this reported deterioration. First, the nursing workload was increased heavily by the Covid‐19 pandemic starting in early 2020 (Nagel et al. 2022). Moreover, the Covid‐19 pandemic has affected the well‐being of nurses and their working conditions (Nagel et al. 2022; Sihvola et al. 2023), which in turn is associated with the quality of care (Sihvola et al. 2023). These factors may have influenced the delivery of fundamental care and the results of this study.

Second, there was an increasing lack of nursing staff and an increase in turnover rates (Tyks 2023) during the period of this study. Organisational turnover is associated with nurse burnout, which leads to reduced staff efficacy (Kelly et al. 2021). Third, in 2021, a nationwide nurses' strike was threatened due to wage negotiations. It may be that the hospital where the study was conducted lacked the capacity to efficiently react to these rapid changes and challenges. When there is a nursing shortage, nurses may need to make decisions about how to prioritise tasks. Fundamental care might have been the target of missed care, which has been further linked to poor staffing and poor patient safety (Cho et al. 2020). Ausserhofer et al. (2014) found that nurses working in hospitals with lower workloads and fewer demands on non‐nursing tasks (e.g., transporting patients within the hospital, cleaning patient rooms and equipment) have lower rates of leaving nursing care undone. Fourthly, the consolidation of the new evidence‐based nursing practices will be successful if long‐term practical support is given by the managers at different levels. For example, previous research shows that transformational leadership increases nursing staff satisfaction and has a positive impact on quality of care and patient outcomes (Robbins and Davidhizar 2020; Gebreheat et al. 2023). It may be that NMs felt overburdened by multiple responsibilities (Julnes et al. 2022) during the Covid‐19 pandemic and the lack of nursing staff. Therefore, they may have been unable to provide adequate support to their staff in establishing best practices, even though the study by Nurmeksela et al. (2021) indicates that organising and developing the unit are the most frequently performed activities of NMs in Finnish acute care hospitals. Moreover, NMs perform clinical work the least (Nurmeksela et al. 2021), likely due to their heavy workload. However, nurse leaders play a key role in ensuring the availability of essential resources for nursing care and in promoting better patient outcomes through a coordinated and collaborative process within the nursing team (Mudd et al. 2023; Ottonello et al. 2023). To cope with demanding managerial tasks, it is important for healthcare organisations to also consider factors that contribute to the job satisfaction and retention of NMs, such as the relationship between managers and staff, the ability of managers to focus on their tasks and the staff support from the organisation and managers (Cox 2019).

The results of this study suggest that the hospital may benefit from continuing to develop fundamental care by using the tools (e.g., minimum criteria for fundamental care, workbooks for keeping records of development) and practices (e.g., project agents, training and workshops) developed in the STEPPI project. These tools and practices serve as a common framework for the delivery of fundamental care in the daily work of nurses and NMs as the shortage of resources for nursing staff continues to highlight the importance of consistent care practices (Ball et al. 2016; Scott et al. 2019). The STEPPI project was started in a hospital as part of a study and, due to the importance of the topic, it has now spread to 17 healthcare, education and research organisations, forming a national network of cooperation in Finland that is coordinated by the hospital in the study. The STEPPI project is currently in its second phase and has added two new areas of fundamental care (interaction and encounter, and privacy) to be developed. The results indicate that, in the future, in addition to these two new areas, it will be important for nursing developers to focus on other areas of fundamental care in line with their own objectives and unit practices, to share good practices within the hospital and nationally and to learn from each other.

The results of this study show a correlation between the quality of fundamental care in different areas as assessed by nurses. The strongest correlations were between areas of respiration and blood circulation, and mobility and postural care. In 2021, the correlation was weaker than in the assessment from 2015. This is somewhat surprising as there were many Covid‐19 patients in 2021. This change may also mean that the respondents implemented fundamental care inconsistently so that the quality has rather deteriorated in some areas and increased in others. It may also be that, as the shortage of the nursing workforce (Ball et al. 2016; Scott et al. 2019) and the turnover of permanent nurses has increased (Tyks 2023), performance has deteriorated in general. It is further possible that work descriptions for substitutes have been unclear and that hospital units have been busy, resulting in inadequate induction due to rapid changes in staff, as occurred during the Covid‐19 pandemic in other healthcare organisations worldwide (Nagel et al. 2022). This is an important observation to which NMs should seek solutions.

In several areas of fundamental care, the PN's assessed the delivery of fundamental care as better than the RN's. This was the case in the areas of skin care, oral hygiene and nutrition in 2015 and in the areas of oral hygiene and wellness of mind in 2021. It appears that PN's delivered fundamental care in these areas more than RN's. This is logical in the sense that, although fundamental care is the responsibility of all nurses in Finland, it is a particularly central and important part of PNs' work (The Finnish Union of Practical Nurses 2023). Moreover, Kitson et al. (2013) state that nurses may prioritise and deliver care differently depending on tasks and working environments. There were also small differences between units. The quality of some fundamental care areas was found to be better in the inpatient wards compared to that of outpatient clinics. This may be explained by the fact that, in the Finnish healthcare system, outpatient care often focuses on specialised care (e.g., diabetes, cardiac or rheumatoid arthritis outpatient clinics) rather than fundamental care because of the short treatment times and the health issue under investigation. Ausserhofer et al. (2014) point out that increasingly sicker patients and shorter treatment times, as well as the increasing emphasis on specialist care, easily leave fundamental care with less attention and value. However, there seem to be a broad consensus on the essential needs of the individual, and it is important to respect and focus on them through nursing interventions in all care settings to ensure patients' physical and psychosocial well‐being (Kitson et al. 2010, 2019; Feo et al. 2018).

The analysis showed that some nurses felt that fundamental care was not part of their job. It was surprising that many areas were not perceived by the NMs as falling within their remit. It may be that they interpreted the question as referring only to the delivery of clinical care rather than to monitoring the quality of care. The quality of fundamental care is very much the responsibility of the NMs and should be systematically monitored and promoted by them (Nurmeksela et al. 2021; Mudd et al. 2023). About 50% of NMs felt that patient cleanliness, oral care aspects, assessment of nutritional status, postural care and pressure ulcer monitoring were not their responsibility. This may be an indication that NMs do not recognise their role as quality controllers of nursing care but rather see their role as more administrative (Nurmeksela et al. 2021). However, Mudd et al. (2023) found that nurse managers are not clear on how to support their staff in providing high‐quality fundamental care. Nurmeksela et al. (2021) states that the managerial activities of NMs are mainly focused on collaboration, human resources management, and operations management, and to a varying extent on clinical care. Structured rounds by NMs increase patient satisfaction and some aspects of the quality of care (Bayram et al. 2023). Such rounds, including aspects of fundamental care, could also be implemented and tested in the studied organisation. Also, Muntlin et al. (2023) highlight, as an example, Gemba Walks, where the NMs observe actual clinical practice and have reflective sessions with nurses. Moreover, NMs can benefit from additional resources and guidance to help them support the delivery of fundamental care in their clinical areas similarly to what Mudd et al. (2023) have suggested.

RNs also conveyed that they did not consider some tasks (e.g., using nutritional risk or pressure ulcer risk assessment instruments) to be part of their work. These issues were similar to the ones reported by the NMs but were not as strongly expressed. It may be that the nurses' heavy workload, an increasing amount of non‐nursing tasks, such as cleaning patient rooms and equipment (Ausserhofer et al. 2014) and an increasing amount of paperwork are distracting nurses from direct patient care. In addition, tasks that have increasingly been transferred from physicians to nurses, such as writing prescriptions, as well as independent nursing consultations have led to a broadening and changing of nurses' job descriptions (Ensio et al. 2019) and possibly dividing time between more tasks. Agreeably, the PNs expressed that all areas of fundamental care were part of their work, which is in line with the PNs' defined area of expertise and job descriptions in Finland (The Finnish Union of Practical Nurses 2023).

The patients' responses showed that their experience of the fundamental care they received was mostly good. This result is in line with the overall feedback collected by the hospital where the study was conducted, with 94% of patients rating the care they received as good (Tyks 2023). Interestingly, many patients did not express a need to deal with issues related to their wellness of mind during their care in the hospital. This may be related to the Finnish culture, where it is generally not common practice to speak openly about one's feelings. This result may also be reflected in short treatment times and short‐term care relationships between nurses and patients in specialised care. This study highlights the nurses' perceptions of the minimal necessity for the assessment of a patient's wellness of mind, as this item scored high as not being a part of their work. This is in line with the patients' expressions. Also, van Belle et al. (2020) report that nurses focus less on the patients' psychosocial and interpersonal needs. Reasons for treatment (e.g., trauma, cancer, Covid‐19 or a new chronic illness) and hospitalisation (e.g., intensive care) generally trigger emotions in people that need to be dealt with (McColl‐Kennedy et al. 2017). Therefore, it is important to give attention to how a patient's wellness of mind is individually best supported. Also, in this study, the patients' suggestions for further improvements regarding care were related to the provision of patient‐centred care. Further research is needed to determine how to best promote this.

## 6 Strengths and Limitations

This study has some limitations. First, the study was conducted in only one hospital, and the response rate remained quite low. The relatively low response rate may be due to the method of data collection and is therefore quite typical, as this study used an online survey. Response rates for online surveys can vary widely. Reasons for a low response rate may include such factors as a lack of reminders, the number of items, the type of questions, or who the researchers are, but we can only speculate about the reason behind the response rate in this study (L'Ecuyer et al. 2023). This is a cause for concern, and new and different ways of collecting data should be considered in the future. However, the hospital in which the survey was conducted is large and encompasses several medical specialities and units, and the respondents represent these medical specialities widely. Although the findings cannot be generalised, they give an overview of the quality of fundamental care at two different points in time and from two perspectives: from nurses and patients. Furthermore, this study cannot fully evaluate the effects of the STEPPI project because several things may have influenced the development and delivery of fundamental care practices during the study period. However, this study can assess the delivery of the 12 areas of fundamental care and the quality of fundamental care practices perceived by the participating nurses at two different time points and reflect on what meaning the project had in this context.

Second, the Quality of Fundamental Care questionnaire for nurses was developed based on nursing research, guidance and classification regarding nursing documentation in Finland and comments from the hospital's nursing development team. Minor linguistic revisions were made to the questionnaire, and a few items were split in 2021 to make the questionnaire more readable and increase its validity. Although editing the questionnaire is a limitation, the changes were minor and have been described and adjusted so that the areas of fundamental care remain comparable between the two data collection timepoints. In addition, the validity of the questionnaire was assessed in 2015 and 2021 by a nursing development team with a wide range of expertise in nursing, leadership and research.

The Cronbach's alpha coefficient was used to test the internal consistency of the questionnaire. The area of Personal hygiene and professionals' aseptic behaviour had the lowest score of 0.58 in 2015, but this was improved to 0.63 in 2021. All other areas of fundamental care had a score above 0.70. Although the questionnaire had face validity and satisfactory internal consistency, the psychometric aspects of the questionnaire need to be further tested.

Third, the Quality of Fundamental Care questionnaire aimed to measure the delivery and documentation of fundamental care as reported by nurses themselves. Self‐assessment is a widely and commonly used method in nursing research. As self‐evaluation is subjective, it is important that in addition to self‐assessment, there are more objective and multidimensional ways of measuring fundamental care (Conroy et al. 2023). In this study, patients' experiences were examined alongside nurses' self‐assessments to confirm the reported perceptions of nurses. The patient questionnaire was deliberately kept short so that patients would complete it. However, in the future it would be important to study patients' experiences of the quality of fundamental care more comprehensively than with merely one question per area.

## 7 Conclusions

Overall, the quality of fundamental care, as perceived by both nursing staff and patients, was high. Despite the long‐term improvements in the quality of fundamental care achieved through the STEPPI project in the hospital, the quality of fundamental care deteriorated during the follow‐up period in general and in specific areas, including skin condition and cleansing, elimination, nutrition and blood circulation, according to the nurses' assessments. In addition, the employees working as RNs or NMs did not feel that all areas of fundamental care were relevant to their work. It may be that, starting in 2020, the Covid‐19 pandemic and a general shortage of nurses in the hospital contributed to nurses having to decide which tasks to postpone and how to prioritise tasks between fundamental and specialised care, which is worrying.

Hence, further development of fundamental care in the hospital is recommended by using the tools and practices developed in the STEPPI project, which serve as a common framework for the delivery of fundamental care in the daily work of nurses and NMs. It is important that nursing developers receive adequate managerial support and focus on all aspects of fundamental care, including the psychosocial and interpersonal needs of patients, in line with the units' own objectives, share good practice at the hospital and national levels and learn from each other. Hospitals are likely to benefit from recurrently done assessments of the quality of fundamental care. Moreover, NMs can benefit from additional resources and guidance to help them support the delivery of fundamental care in their clinical areas, similarly to what Mudd et al. (2023) suggest.

Further studies should be planned to systematically assess the quality and effectiveness of fundamental care using mixed‐methods approaches, from the perspective of nurses, patients and their families. Future studies should consider more targeted approaches by refining data collection methods and inclusion and exclusion criteria to accurately reflect the objectives of more robust study designs. Research is also needed to explore why some nurses in this study expressed that key aspects of fundamental care were not part of their work, and to examine the relationships between fundamental care, patient outcomes and costs.

## Author Contributions

H.‐S.K., L.‐M.P., A.‐S.K., S.S., P.S. and A.H. contributed to the conception and design, interpretation of data, manuscript preparation and critical revision of the manuscript. H.‐S.K., A.‐S.K., P.S. and A.H. contributed to the acquisition of data. M.P. contributed to the analysis of the data and manuscript preparation.

## Disclosure

Statistics: The quantitative data has been analysed by statistician Miko Pasanen and he is on the author team.

## Conflicts of Interest

The authors declare no conflicts of interest.

## Data Availability

The data cannot be shared for confidentiality matters.
